# Profiling gut microbiome dynamics in subacute thyroiditis: Implications for pathogenesis, diagnosis, and treatment

**DOI:** 10.1515/med-2025-1291

**Published:** 2025-10-15

**Authors:** Jiayun Li, Jiasheng Ju, Qian Xu, Xiang Han, Haibing Ju

**Affiliations:** Department of Endocrinology, 920th Hospital of the Joint Logistics Support Force of the Chinese People’s Liberation Army, Kunming, Yunnan, 650032, China; Department of Endocrinology, Huize County People’s Hospital, Qujing, Yunnan, 654200, China; Department of Neurosurgery, Zhujiang Hospital of Southern Medical University, Guangzhou, Guangdong, 510282, China

**Keywords:** subacute thyroiditis, gut microbiota, 16S ribosomal RNA, prednisone

## Abstract

**Aim:**

The aim of this study is to characterize gut microbiome alterations in newly diagnosed subacute thyroiditis (SAT) patients, and identify potential microbial signatures associated with SAT and treatment response.

**Methods:**

Fecal samples collected from 20 newly diagnosed SAT patients and 20 healthy controls were analyzed using 16S ribosomal RNA gene sequencing. Bioinformatics analysis was performed to assess alpha and beta diversity, taxonomic composition, and differential abundance of gut microbiota between the groups. Correlations between gut microbiome and clinical parameters were also investigated.

**Results:**

Newly diagnosed SAT patients exhibited significant alterations in gut microbiota composition. There was increased abundance of *Escherichia-Shigella*, *Bifidobacterium*, *Akkermansia*, *Veillonella*, and *Streptococcus*, while the abundance of *Bacteroidetes*, *Faecalibacterium*, *Prevotella*, *Roseburia*, and *Ruminococcus* were significantly decreased. Prednisolone treatment partially normalized the gut microbiota, with *Lactobacillus*, *Lactobacillaceae*, *Lactobacillus reuteri*, and *Prevotella* emerging as key biomarkers in post-treatment SAT. Significant correlations were found between specific gut microbiome and clinical markers.

**Conclusion:**

SAT is associated with distinct gut microbiome alterations, partially reversible with treatment, which suggest a potential role for the gut microbiome in SAT pathogenesis and treatment response.

## 1 Introduction

The gut microbiome, a complex and diverse microbial ecosystem, is often referred to as the “second genome” due to its significant impact on human physiology. These microorganisms play pivotal roles in various physiological processes, including nutrient metabolism, immune modulation, endocrine system homeostasis, and neurodevelopment [1]. The thyroid gland’s embryonic origin in the primitive foregut [2], a shared developmental origin with the gastrointestinal system, suggests a potential relationship between thyroid disorders and gut microbiome dysbiosis. This developmental link underscores the importance of investigating the intricate relationship between these two systems to elucidate the role of gut microbiome in thyroid pathophysiology.

Advancements in sequencing technology have revolutionized the study of the gut microbiota by enabling the identification and quantification of bacterial populations at an unprecedented level of detail. This has unlocked novel insights into the intricate interplay between gut microbiome and human disease conditions. In 2017, Lerner et al. [3] provided compelling evidence for a potential pathogenic role of the gut microbiome in primary hypothyroidism, introducing the concept of the “thyroid-gut axis” [4] and highlighting the intricate relationship between these two systems. Subsequent studies have confirmed associations between autoimmune thyroid diseases [5,6,7,8], such as Graves’ disease (GD) and Hashimoto’s thyroiditis (HT), and dysbiosis in the gut microbiome.

Subacute thyroiditis (SAT) is the most prevalent self-limiting form of thyroiditis, characterized by thyroidal pain, systemic inflammation, and thyrotoxicosis. However, its precise etiology and pathogenesis remain incompletely understood [9], and the potential contribution of the gut microbiome to this condition has not been fully elucidated. The aim of this study is to address this knowledge gap by profiling the gut microbiota composition in SAT patients compared to healthy controls. Additionally, we sought to investigate dynamic changes in the gut microbiome before and after prednisolone treatment, and to assess potential correlations between these alterations and relevant clinical parameters. By identifying distinct microbial signatures associated with SAT, this research aims to provide insights into the underlying pathophysiology of the disease and potentially pave the way for the development of novel diagnostic biomarkers, personalized therapeutic interventions, and improved prognostication for SAT patients.

## 2 Materials and methods

### 2.1 Participant selection and enrollment

Inclusion criteria for the SAT cohort included a diagnosis of SAT according to the “Chinese Guidelines for the Diagnosis and Treatment of Thyroid Diseases: Thyroiditis”, age between 25 and 40 years, Han ethnicity, body mass index (BMI) between 18 and 28 kg/m^2^, and no history of other thyroid, autoimmune, endocrine, or metabolic disorders. Additionally, individuals with no recent history of diarrhea or antibiotic/probiotic use within 1 month prior to stool sample collection were included.

Exclusion criteria were a history of using antibiotics, hormones (excluding thyroid hormone replacement), laxatives, herbal medicines, or microbial preparations within 3 months prior to enrollment. Individuals with hypertension, diabetes mellitus, dyslipidemia, or a BMI outside the normal range were also excluded. Further exclusion criteria included a history of malignancy, gastrointestinal disease, or gastrointestinal surgery, as well as pregnancy, lactation, smoking, or alcohol consumption. Finally, individuals with other chronic inflammatory conditions were not eligible for participation.

Twenty newly diagnosed, treatment-naïve SAT patients (8 men, 12 women) with a mean age of 45.37 ± 10.53 years and a mean BMI of 24.17 ± 2.23 kg/m², presenting to the Endocrinology Department of the 920th Hospital of the Joint Logistics Support Force of the People’s Liberation Army of China between June 2022 and March 2023, were enrolled as the experimental group, and were treated with oral prednisone (0.5–1 mg/kg/day) for 6–8 weeks. Concurrently, 20 healthy individuals (9 men, 11 women) with a mean age of 48.24 ± 11.33 years and a mean BMI of 24.45 ± 2.56 kg/m², undergoing routine physical examinations during the same period, were recruited as the control group.

### 2.2 Sample collection and processing

Stool samples were collected from a total of 60 participants, comprising 20 SAT patients before treatment, 20 SAT patients after 12 weeks of treatment, and 20 healthy control subjects. All samples were immediately stored at −80°C. Blood samples were also obtained from SAT patients before and after treatment for subsequent analysis.

### 2.3 DNA extraction, amplification, and library preparation

Microbial genomic DNA was extracted from all stool samples using a modified cetyltrimethylammonium bromide protocol optimized for fecal material. DNA purity and integrity were assessed using spectrophotometry and agarose gel electrophoresis. The extracted metagenomic DNA served as the template for multiplex PCR and high-fidelity low-cycle PCR amplification. Amplicons were purified using AmPure XT beads (Beckman Coulter, CA) following the manufacturer’s instructions. Purified amplicons were quantified, normalized, and pooled in equimolar ratios to create the final sequencing library.

### 2.4 Sequencing and data processing

The DNA libraries were assessed for quality and quantity using an Agilent 2100 Bioanalyzer and Illumina library quantification kit. Libraries with a concentration of 2 nM or higher were considered suitable for sequencing. Paired-end sequencing (2 × 250 base pairs) was performed on a NovaSeq 6000 platform. Raw sequencing data underwent quality filtering, adapter trimming, and chimera removal using established pipelines.

### 2.5 Bioinformatics analysis

Amplicon Sequence Variants (ASVs) were inferred using Qiime dada2 denoise-paired and served as the operational taxonomic units for downstream analysis. Taxonomic classification and annotation of ASVs were performed against the SILVA 16S rRNA gene database. Alpha diversity and beta diversity metrics were calculated to assess microbial community richness, evenness, and composition. Differential abundance analysis was conducted to identify taxa significantly associated with SAT status and treatment response.

### 2.6 Statistical analysis

Statistical analyses and visualization were performed using R (Version 4.2.1), Origin (Version 2021b), and SPSS (Version 26.0). Spearman correlation analysis was used to assess the relationship between gut microbiome composition and clinical indicators. For normally distributed data, results were presented as mean value ± standard deviation (SD). Independent *t*-tests were used for between-group comparisons (SAT vs. control), paired *t*-tests for within-group comparisons before and after treatment, and one-way analysis of variance followed by Tukey’s honestly significant difference test for multiple group comparisons. Non-normally distributed data were presented as median and interquartile range and compared using the Mann-Whitney U test for two groups and the Kruskal-Wallis test for multiple groups. Mann-Whitney U *p*-values were adjusted for multiple comparisons via Benjamini-Hochberg false discovery rate (FDR) correction control. Significance was defined as *q*-value <0.05.


**Ethical approval**: The study protocol was approved by the Medical Ethics Committee of the 920th Hospital of the Joint Logistics Support Force of the People’s Liberation Army of China. This study was conducted in accordance with the declaration of Helsinki and approved by the Ethics Committee of the 920th Hospital of the Joint Logistics Support Force of the Chinese People’s Liberation Army (No. 2022-128-01).
**Informed consent:** All participants provided written informed consent before enrollment.

## 3 Results

### 3.1 Prednisone normalizes thyroid function and suppresses inflammation in SAT patients

After treatment with oral prednisone (0.5–1 mg/kg/day) for 6–8 weeks, significant reductions (*P* < 0.05) were observed in serum concentrations of tri-iodothyronine (T3), thyroxine (T4), free tri-iodothyronine (FT3), free thyroxine (FT4), thyroglobulin (Tg), thyroid peroxidase antibody (TPO), C-reactive protein (CRP), erythrocyte sedimentation rate (ESR), white blood cell count (WBC), neutrophil (NEU) count, ferritin (FT), and fibrinogen (FIB), while thyroid-stimulating hormone (TSH) levels increased significantly (*P* < 0.05) (Table 1). These results indicate that prednisone effectively reduces both thyroid hormone production and the inflammatory response in SAT patients.

**Table 1:** Comparison of clinical and laboratory parameters in SAT patients before and after treatment

	Control value	Before treatment	After treatment	*z*-value	*p*-value
T3 (nmol/L)	1.01–2.96	3.25 (2.79, 4.47)	1.92 (1.56, 2.07)	−3.36	0.001*
T4 (nmol/L)	55.4–161.25	204.80 (158.55, 237.03)	112.90 (92.78, 129.63)	−3.31	0.001*
TSH (nmol/L)	0.38–4.34	0.01 (0.01, 0.08)	2.00 (0.69, 2.87)	−3.36	0.001*
FT3 (nmol/L)	2.77–6.31	14.30 (9.77, 26.84)	4.87 (4.79, 6.33)	−3.52	0.000*
FT4 (nmol/L)	10.44–24.38	38.49 (29.45, 46.10)	15.38 (13.83, 19.04)	−3.516	0.000*
Tg (nmol/L)	0–20	20.59 (15.32, 26.88)	16.83 (8.72, 18.65)	−2.068	0.039*
TgAb (IU/mL)	0–2.1	1.90 (0.79, 3.70)	2.06 (1.10, 8.02)	−1.112	0.266
TPO (IU/mL)	0–2.1	4.18 (2.79, 8.29)	1.92 (0.94, 2.74)	−3.103	0.002*
CRP (mg/L)	0.0–8.2	36.80 (20.35, 105.30)	3.84 (2.56, 7.98)	−3.413	0.001*
ESR (mm/H)	Male <20	100.00 (50.75, 117.00)	4.00 (3.25, 19.50)	−3.465	0.001*
	Female <34				
WBC (10^9^/L)	3.50–9.50	9.92 (5.10, 18.56)	6.64 (5.91, 7.58)	−2.414	0.006*
Neu (10^9^/L)	1.80–6.30	5.49 (3.98, 6.91)	3.71 (3.39, 4.70)	−2.603	0.015*
Lym (10^9^/L)	1.10–3.20	1.72 (1.28, 2.12)	1.94 (1.74, 2.30)	−1.655	0.098
UA (μmol/L)	Male: 208–428	318.00 (264.25, 412.50)	284.50 (248.00, 341.00)	−1.034	0.301
	Female: 155–357				
FT (ng/mL)	18.2–341.2	5.91 (4.36, 7.41)	2.43 (1.97, 3.24)	−3.206	0.001*
FIB (g/L)	150–350	425.60 (389.15, 599.500)	155.60 (51.02, 236.90)	−2.844	0.004*
Glu (mmol/L)	3.89–6.11	5.24 (4.76, 7.27)	5.12 (4.61, 7.26)	−0.259	0.796
TG (mmol/L)	0–1.7	1.38 (0.80, 1.86)	1.84 (1.11, 3.46)	−1.448	0.148
TC (mmol/L)	0–5.2	4.01 (3.13, 4.72)	4.09 (2.98, 5.45)	−0.233	0.816

This table presents the mean values and SD of various clinical and laboratory parameters in SAT patients before and after prednisone treatment. T3: Triiodothyronine; T4: Thyroxine; TSH: Thyroid stimulating hormone; FT3: Free triiothyronine; FT4: Free thyroxine; Tg: Thyroglobulin; TgAb: Antithyroglobulin antibody; TPO: Antithyroid peroxidase antibody; CRP: C-reactive protein; ESR: Blood sedimentation; WBC: White blood cell; NEU: Neutrophils; Lym: Lymphocytes; UA: Uric acid; FT: Ferritin; FIB: Fibrinogen; Glu: Glucose; TG: Triglycerides; TC: Cholesterol. *Indicates *p* < 0.05.

### 3.2 Gut microbiota diversity analysis

#### 3.2.1 Alpha diversity

Newly diagnosed SAT patients exhibited a significantly higher Chao1 index, indicating increased microbial richness, compared to the healthy control group. The Shannon index, indicative of microbial diversity, and Pielou’s evenness index, measuring the uniformity of species distribution, were significantly lower in SAT patients, suggesting reduced diversity and evenness in their gut microbiota (Table 2). This indicates that while SAT patients harbor a greater number of microbial species, their gut microbiota is less diverse and more unevenly distributed. Although not statistically significant (*p* > 0.05), a trend toward decreasing richness and increasing diversity and evenness was observed following prednisone treatment, suggesting a potential shift in microbial composition toward a healthier profile.

**Table 2:** Alpha diversity indices of gut microbiota in SAT patients before and after treatment, and healthy controls

	Healthy control	Before treatment	After treatment	*q* ^1^-value	*Q* ^2^-value	*q* ^3^-value
Chao1	290.48 ± 71.22	299.48 ± 73.01	268.81 ± 75.20	0.34	0.65	0.64
Shannon	5.44 ± 0.68	4.57 ± 1.17	4.96 ± 0.92	0.48	0.09	0.34
Pielou	0.62 ± 0.12	0.57 ± 0.11	0.63 ± 0.07	0.11	0.05	0.26

Data are presented as mean values ± SD. *q* ^1^: *q*-value comparing pre-treatment SAT patients with post-treatment SAT patients; *q* ^2^: *q*-value comparing pre-treatment SAT patients with healthy controls; *q* ^3^: *q*-value comparing post-treatment SAT patients with healthy controls. FDR *q*-value < 0.05.

#### 3.2.2 Beta diversity

**Figure 1:**
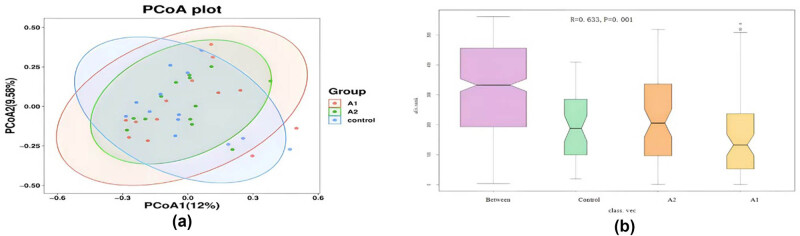
(a) Principal coordinate analysis (PCoA) of unweighted UniFrac distances in gut microbiota. This plot illustrates the distinct clustering of gut microbial communities among the three groups: Pre-treatment SAT patients (A1), post-treatment SAT patients (A2), and healthy control group (Control); (b) ANOSIM results for gut microbiota composition. This diagram displays the results of the ANOSIM analysis (*R* = 0.633, *p* = 0.001), quantifying the dissimilarity between microbial communities in pre-treatment SAT (A1), post-treatment SAT (A2), and healthy controls (Control).

Unweighted unique fraction metric (UniFrac) analysis, a phylogenetic distance metric, revealed distinct clustering of gut microbiota profiles between SAT patients (both pre- and post-treatment) and healthy controls, as visualized along both principal coordinate analysis (PCoA) axes 1 and 2. This separation indicates significant differences in the overall microbial community composition between the groups. Notably, samples within each group clustered tightly together, suggesting greater homogeneity in species composition within groups compared to between them (Figure 1a). Analysis of similarities (ANOSIM) revealed statistically significant group dissimilarities (*R* = 0.633, *p* = 0.001), indicating higher variation between groups compared to within groups (Figure 1b).

### 3.3 Phylum-level composition and differential abundance analysis of gut microbiota

The gut microbiota composition in SAT patients significantly differed from that of healthy controls, particularly in the relative abundance of key phyla. Relative abundance analysis of the 30 most abundant bacterial taxa, classified at the phylum level, revealed that *Firmicutes*, *Bacteroidota*, *Proteobacteria*, *Actinobacteriota*, and *Verrucomicrobiota* were the predominant phyla, constituting over 98% of the total gut microbiota in both healthy controls and SAT patients. Compared to healthy controls, newly diagnosed SAT patients exhibited a significant decrease in the relative abundance of *Firmicutes* and *Verrucomicrobiota* (*p* < 0.05). This depletion of *Firmicutes*, typically associated with maintaining gut homeostasis, and *Verrucomicrobiota*, known for anti-inflammatory properties, could contribute to the dysregulated immune response and inflammation seen in SAT. Following prednisone treatment, a significant increase in *Verrucomicrobiota* was observed (*p* < 0.05), suggesting a potential restoration of beneficial microbes that could contribute to the resolution of inflammation (Table 3).

**Table 3:** Relative abundance of gut microbiota at the phylum level in SAT patients before and after treatment, and healthy controls

	Healthy control	Before treatment	After treatment	*t* ^1^-value	*t* ^2^-value	*q* ^1^-value	*q* ^2^-value
*Firmicutes*	63.33 ± 4.11	49.09 ± 6.37	58.66 ± 7.62	−2.45	1.21	0.04^*^	0.27
*Bacteroidota*	22.33 ± 5.00	13.10 ± 5.12	14.35 ± 5.30	−1.50	0.15	0.18	0.88
*Proteobacteria*	8.49 ± 4.12	19.58 ± 8.66	15.72 ± 4.18	1.10	−0.48	0.30	0.65
*Actinobacteriota*	4.65 ± 1.22	10.41 ± 4.39	10.34 ± 3.85	1.27	−0.10	0.25	0.99
*Verrucomicrobiota*	0.23 ± 0.22	0.07 ± 0.01	0.10 ± 0.01	−1.72	4.31	0.004^*^	0.03^*^

Data are presented as mean values ± SD. Statistical comparisons were performed using *t*-tests, with * indicating significant differences (*q* < 0.05). *t* ^1^ and *q* ^1^ represent the *t*-value and *q*-value comparing pre-treatment SAT patients with healthy controls, while *t* ^2^ and *q* ^2^ represent the *t*-value and *q*-value comparing post-treatment SAT patients with pre-treatment SAT patients.

### 3.4 Genus-level composition and differential abundance analysis of gut microbiota

Differential abundance analysis, using the Mann-Whitney U test, identified significant shifts in the relative abundance of specific bacterial genera in SAT patients compared to healthy controls. Newly diagnosed SAT patients exhibited significant enrichments in *Escherichia-Shigella*, *Bifidobacterium*, *Akkermansia*, *Lactobacillus*, and *Veillonella* (*q* < 0.05), while *Prevotella*, *Faecalibacterium*, *Streptococcus*, *Enterococcus*, and *Clostridium* were significantly depleted (*q* < 0.05). Following SAT treatment, a reversal of some of these trends was observed, with significant increases in *Streptococcus*, *Enterococcus*, *Ruminococcus*, *Faecalibacterium*, *Rothia*, and *Clostridium* and concomitant decreases in *Escherichia-Shigella*, *Bacteroides*, *Lactobacillus*, *Akkermansia*, and *Veillonella* (*q* < 0.05) (Table 4). These findings highlight the dynamic nature of the gut microbiota in SAT patients and its potential responsiveness to prednisone treatment.

**Table 4:** Relative abundance of gut microbiota at the genus level in SAT patients before and after treatment, and healthy controls

	Healthy control	Before treatment	After treatment	*t* ^1^-value	*t* ^2^-value	*q* ^1^-value	*q* ^2^-value
*Bacteroidota*	17.94 ± 2.60	10.18 ± 1.40	11.79 ± 1.80	−10.66	0.401	0.000^*^	0.207
*Faecalibacterium*	14.32 ± 1.19	6.86 ± 1.29	6.98 ± 1.50	−9.58	0.21	0.000^*^	0.84
*Escherichia- Shigella*	7.31 ± 1.74	15.47 ± 1.46	9.67 ± 1.99	15.14	−5.23	0.000^*^	0.001^*^
*Bifidobacterium*	3.39 ± 0.49	9.55 ± 0.45	8.9 ± 0.62	29.54	−1.848	0.000^*^	0.107
*Lachnospiraceae*	3.53 ± 1.08	4.58 ± 1.7	4.47 ± 1.19	0.487	−0.086	0.641	0.934
*Agathobacter*	7.72 ± 1.26	8.42 ± 1.15	2.80 ± 0.77	0.16	−13.76	0.877	0.000^*^
*Streptococcus*	1.53 ± 0.08	0.42 ± 0.07	2.16 ± 0.36	−8.009	4.607	0.000^*^	0.002^*^
*Ruminococcus gnavus*	1.36 ± 0.77	5.98 ± 1.01	3.64 ± 1.93	2.97	−2.3	0.039^*^	0.048^*^
*Subdoligranulum*	1.59 ± 0.16	1.05 ± 0.15	0.98 ± 0.17	−2.277	−0.225	0.057	0.828
*Dialister*	2.92 ± 0.23	0.11 ± 0.003	2.76 ± 0.005	−2.264	2.305	0.038^*^	0.023^*^
*Megamonas*	2.83 ± 0.64	0.04 ± 0.002	0.04 ± 0.022	−2.69	0.17	0.034^*^	0.87
*Prevotella*	1.83 ± 0.79	0.37 ± 0.09	0.13 ± 0.02	−4.64	−0.665	0.002^*^	0.527
*Roseburia*	17.94 ± 2.60	0.94 ± 0.10	1.07 ± 0.16	−2.918	0.708	0.022^*^	0.502
*Ruminococcus*	14.32 ± 1.19	0.61 ± 0.023	5.16 ± 1.09	−4.63	9.76	0.002^*^	0.000^*^
*Coprococcus*	7.31 ± 1.74	0.445 ± 0.016	3.99 ± 0.62	−2.96	15.67	0.021^*^	0.000^*^
*Akkermansia muciniphila*	3.39 ± 0.49	5.82 ± 1.15	0.10 ± 0.001	6.87	−7.601	0.000^*^	0.000^*^
*Lactobacillus reuteri*	3.53 ± 1.08	0.53 ± 0.03	2.37 ± 0.15	−1.35	5.89	0.216	0.001^*^
*Veillonella*	7.72 ± 1.26	1.95 ± 0.40	0.20 ± 0.03	−3.91	−14.30	0.006^*^	0.000^*^
*Lachnoclostridium*	0.15 ± 0.004	0.044 ± 0.006	0.21 ± 0.01	−0.73	4.73	0.753	0.000^*^

Data are presented as mean value ± SD. Statistical comparisons were performed using *t*-tests, with * indicating significant differences (*q* < 0.05). *t* ^1^ and *q* ^1^ represent the *t*-value and *q*-value comparing pre-treatment SAT patients with healthy controls, while t^2^ and *q* ^2^ represent the *t*-value and *q*-value comparing post-treatment SAT patients with pre-treatment SAT patients.

### 3.5 Identification of microbial biomarkers

**Figure 2:**
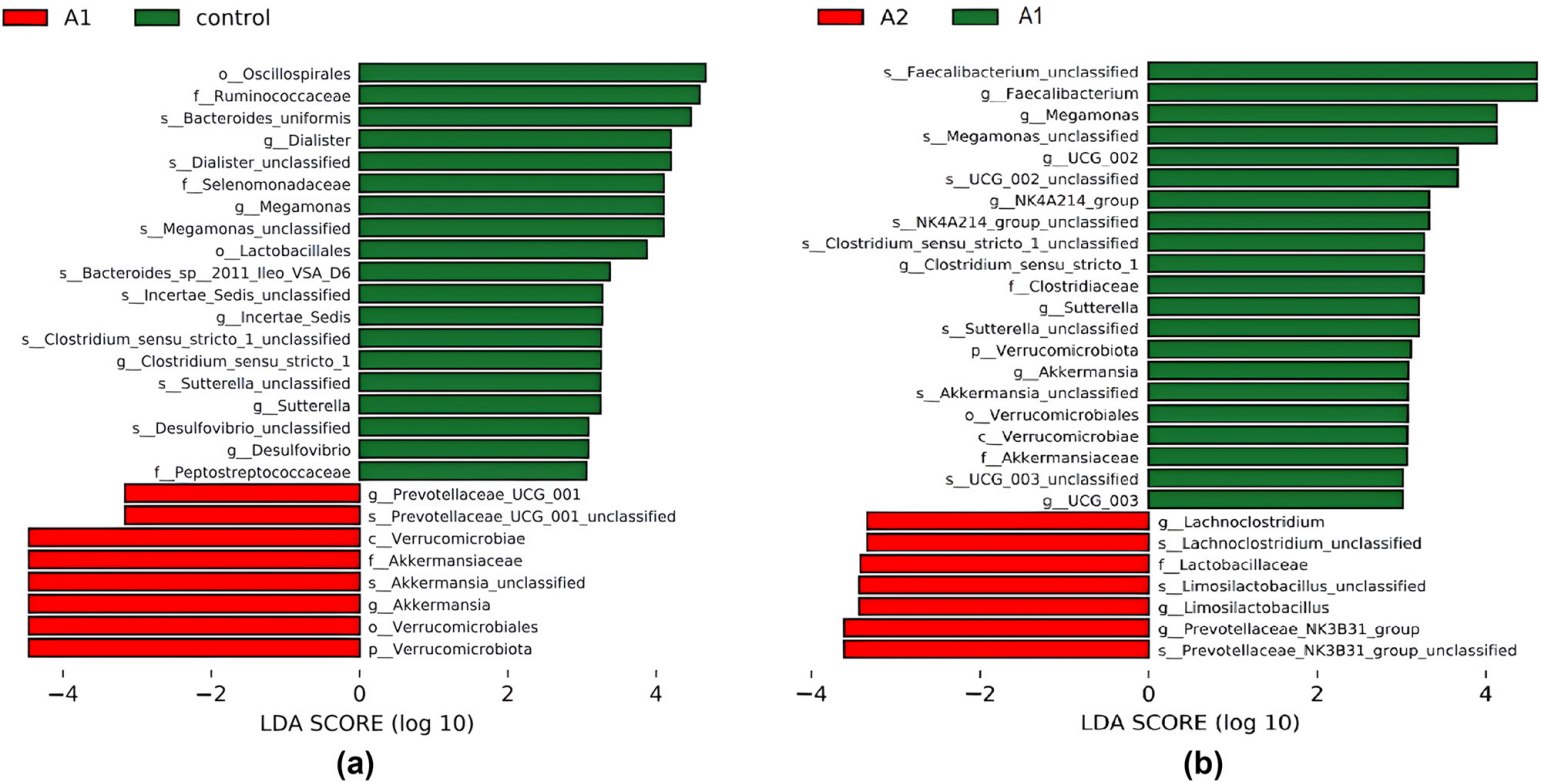
(a) Histogram of LEfSe scores for differentially abundant taxa in pre-treatment SAT patients and healthy controls. This histogram displays the distribution of LDA scores for bacterial taxa identified as significantly differentially abundant between pre-treatment SAT patients (A1) and healthy controls (Control); (b) histogram of LEfSe scores for differentially abundant taxa in post-treatment and pre-treatment SAT patients. This histogram displays the distribution of LDA scores for bacterial taxa identified as significantly differentially abundant between post-treatment SAT patients (A2) and pre-treatment SAT patients (A1). The LDA score quantifies the magnitude and direction of the effect (positive for enrichment in A2, negative for enrichment in A1).

Linear discriminant analysis (LDA) effect size (LEfSe) analysis revealed distinct microbial signatures associated with SAT and its treatment response. Nineteen taxa were found to be enriched in healthy controls, while eight taxa were specifically enriched in SAT patients, with *Prevotella* and *Akkermansia* emerging as key discriminative biomarkers (Figure 2a). Interestingly, the microbial signature shifted following prednisone treatment. Twenty-one taxa were identified as biomarkers in the pre-treatment group, whereas only seven were identified in the post-treatment group, including *Ruminococcus*, *Rothia*, and *Prevotella* (Figure 2b). These findings underscore the distinct microbial signatures associated with SAT and its treatment response, highlighting the potential of these taxa as biomarkers for monitoring therapeutic efficacy and elucidating the dynamic shifts in gut microbiota composition associated with thyroid disorders.

### 3.6 Correlation analysis between gut microbiota and clinical parameters in SAT patients

**Figure 3:**
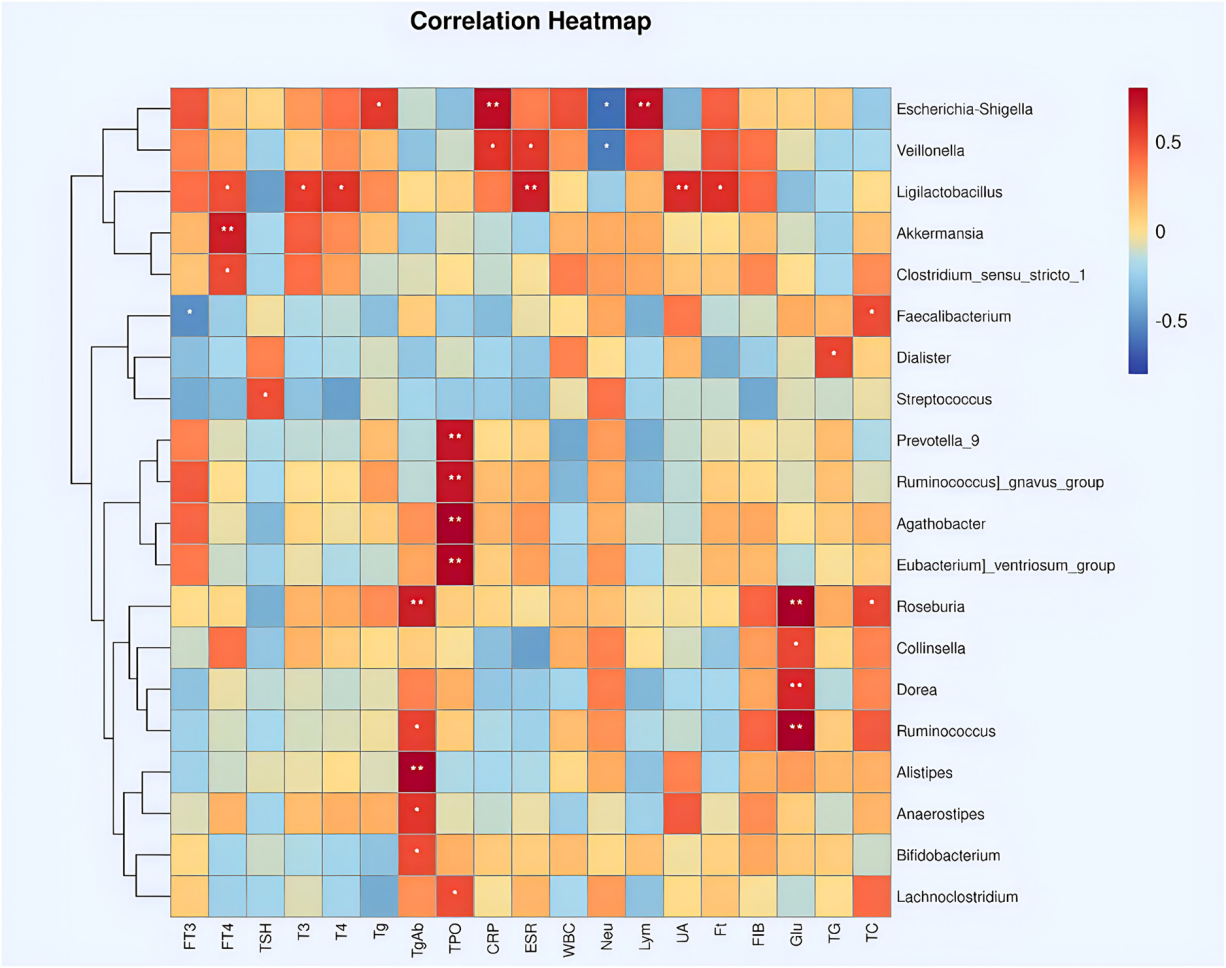
Heatmap of spearman correlation analysis between gut microbiota and clinical parameters in SAT patients. This heatmap illustrates the strength and direction of correlations between the relative abundance of specific gut microbial taxa (rows) and clinical parameters (columns) in SAT patients. Positive correlations are represented by warm colors (red), negative correlations by cool colors (blue), and non-significant correlations by white. The intensity of the color reflects the magnitude of the correlation coefficient. *P < 0.05, **P < 0.01.

Spearman correlation analysis revealed significant associations between the relative abundance of specific gut microbial taxa and clinical parameters in SAT patients, suggesting a potential interplay between gut microbiota and thyroid function and inflammation. *Ruminococcus* abundance was negatively correlated with FT4 and ESR, and positively correlated with Tg. *Streptococcus* abundance exhibited negative correlations with CRP, total T4, FIB, and TPO. *Actinomyces* abundance was negatively correlated with Tg and thyroglobulin antibody (TgAb). *Romboutsia* abundance exhibited negative correlations with CRP and total cholesterol (TC). *Enterobacter* abundance was negatively correlated with WBC, T3, T4, FT3, TPO, and FIB, and positively correlated with FT. *Bacteroides* abundance was negatively correlated with FIB, FT3, and T3, and positively correlated with TSH and Tg. *Lactobacillus* abundance was positively correlated with lymphocytes (Lym) and Tg, while *Enterococcus* abundance was negatively correlated with ESR. Additionally, the abundance of *Bifidobacterium*, *Roseburia*, *Bacteroides*, and *Faecalibacterium* demonstrated positive correlations with TgAb and TPO (Figure 3).

These findings reveal potential associations between specific gut microbial taxa and clinical parameters in SAT patients, suggesting their potential relevance as biomarkers in the assessment and management of thyroid-related conditions.

## 4 Discussion

Our study investigated the gut microbiome of patients with SAT before and after prednisone treatment, revealing a distinct microbial signature associated with the disease. Notably, SAT patients exhibited reduced microbial diversity and enrichment of specific taxa such as *Prevotella* and *Akkermansia*, correlating with altered thyroid function and elevated inflammatory markers. This suggests a potential role for gut dysbiosis in SAT pathogenesis. Importantly, prednisone treatment not only ameliorated the clinical manifestations of SAT but also shifted the gut microbiome toward a healthier state, as evidenced by the increased abundance of beneficial bacteria such as *Verrucomicrobiota*. These findings underscore the intricate interaction between the gut microbiome and thyroid health, highlighting potential avenues for novel diagnostic and therapeutic strategies in SAT.

Consistent with previous studies, our findings indicate that SAT is associated with elevated levels of inflammatory markers, including FT, FIB, ESR and CRP. FT is an acute-phase reactant that increases in response to systemic inflammation. Previous studies in patients with SAT have reported significant decreases in FT, ESR, and CRP levels following treatment, with a positive correlation observed between FT and both ESR and CRP [10]. FIB, another acute-phase protein, is also elevated in SAT and decreases as the condition improves [11]. Our study further substantiates these findings by demonstrating that newly diagnosed SAT patients exhibit elevated levels of T3, T4, FT3, FT4, Tg, TPO, CRP, ESR, WBC, NEU count, FT, and FIB. Following prednisone treatment, these biomarkers significantly decreased, consistent with the expected resolution of inflammation. This observation underscores the potential of FT and FIB, in addition to ESR and CRP, as inflammatory biomarkers that reflect the severity of SAT and enable the monitoring of disease activity and response to treatment.

While research on the gut microbiome in SAT is limited, studies on other thyroid disorders such as GD and HT provide context for interpreting our findings. In GD, studies consistently report enrichment of genera such as *Bifidobacterium* and *Lactobacillus*, with concomitant depletion of *Blautia* and *Ruminococcus* [5,6]. In contrast, research in HT reveals increased abundance of *Blautia*, *Roseburia*, *Ruminococcus*, *Dialister*, *Sporobacter*, and *Haemophilus*, alongside reductions in *Bifidobacterium* and *Prevotella* [7,8]. Our study findings demonstrate that newly diagnosed SAT patients exhibit a distinct gut microbiota profile characterized by significant increases in the abundance of genera such as *Escherichia-Shigella*, *Bifidobacterium*, *Akkermansia*, *Veillonella*, and *Enterococcus*, accompanied by notable decreases in genera such as *Bacteroides*, *Faecalibacterium*, *Streptococcus*, *Veillonella*, and *Ruminococcus*. Following prednisone treatment, this profile shifts, with significant increased abundance in *Streptococcus*, *Enterococcus*, *Ruminococcus*, *Faecalibacterium*, *Roseburia*, and *Lactobacillus*, and decreased abundance in *Escherichia-Shigella*, *Bacteroides*, *Veillonella*, *Akkermansia*, and *Enterococcus*.

Correlation analyses revealed distinct associations between specific bacterial taxa and clinical parameters in SAT patients, highlighting their potential roles in disease pathogenesis and treatment response. Key biomarkers identified in newly diagnosed SAT patients include *Prevotella* and *Akkermansia*, while post-treatment biomarkers comprise *Faecalibacterium*, *Roseburia*, and *Prevotella*. *Escherichia-Shigella* abundance correlates positively with Tg, CRP, and Lym, but negatively with NEU count. *Veillonella* abundance correlates positively with CRP, ESR, and the neutrophil-to-lymphocyte ratio (NLR), and negatively with NEU count. *Lactobacillus* abundance exhibits positive correlations with FT4, total T3, total T4, ESR, NLR, uric acid (UA), and FT. *Akkermansia* abundance positively correlates with FT4, while *Faecalibacterium* abundance correlates positively with TC and negatively with FT3. *Enterococcus* abundance positively correlates with Tg, and *Streptococcus* abundance with TSH. *Prevotella*, *Veillonella*, *Faecalibacterium*, *Enterococcus*, and *Ruminococcus* abundances demonstrate positive correlations with TPO. *Roseburia* abundance correlates positively with TgAb, glucose (Glu), and TC. *Collinsella*, *Dorea*, and *Ruminococcus* abundances correlate positively with Glu, while *Actinomyces*, *Faecalibacterium*, and *Bifidobacterium* abundances positively correlate with TgAb.

*Prevotella*, known for its anti-inflammatory properties due to the production of metabolites such as propionic acid [12], has been shown to suppress T helper 17 (Th17) polarization and promote anti-inflammatory regulatory T cells (Treg)/T regulatory type 1 (Tr1) cell differentiation in the gut [13,14]. Consistent with findings in other autoimmune diseases, such as HT [15], type 1 diabetes [16], and multiple sclerosis [17], we observed a decreased relative abundance of *Prevotella* in SAT patients. This depletion, which was positively correlated with TPO levels, suggests a potential contribution to the inflammatory and autoimmune processes in SAT. Excessive proliferation of *Akkermansia* can disrupt the intestinal barrier integrity and exacerbate inflammation [18]. Our study revealed a significant enrichment of *Akkermansia* in SAT patients, coupled with decreased abundances of commensal bacteria such as *Faecalibacterium* and *Roseburia*. This dysbiosis may contribute to reduced species diversity, intestinal barrier dysfunction, and ultimately, the progression of SAT.

*Ruminococcus*, an important gut commensal, ferments polysaccharides into short-chain fatty acids (SCFAs) such as butyrate and acetate [19]. These SCFAs promote intestinal epithelial cell growth, enhance gut barrier function, and modulate immune responses [20]. As a biomarker enriched in the post-treatment SAT group, *Ruminococcus* may play a crucial role in restoring gut microbial balance and facilitating recovery from SAT. Additionally, *Veillonella*, by metabolizing lactate into SCFAs, regulates SCFA and lactate dynamics, contributing to the maintenance of normal gut function [21,22,23].

Glucocorticoids (GCs), primary stress hormones with anti-inflammatory and immunosuppressive properties, are known to influence gut microbiota composition. Yun [24] demonstrated that prednisolone treatment in rats increased the relative abundance of *Proteobacteria* and *Bacillus*, while decreasing *Bacteroides*, *Prevotella*, and *Lactobacillus*. Similarly, a study by Lauren [25] in red squirrels (*Tamiasciurus hudsonicus*) revealed a negative correlation between host GC levels and gut microbiota alpha diversity. Elevated GC levels were associated with increased abundance of *Enterobacteriaceae*, *Streptococcus*, *Enterococcus*, *Prevotella*, *Ruminococcus*, *Clostridium*, *Treponema*, and *Megasphaera*, and decreased abundance of *Yersinia* and *Salmonella*.

In our study, all SAT patients received glucocorticoid therapy, a factor that may have influenced the observed changes in gut microbiota composition. Specifically, we noted decreased microbial richness and increased diversity at the phylum level, characterized by an increase in *Firmicutes*, *Bacteroidetes*, and *Verrucomicrobia*, and a decrease in *Actinobacteria* and *Proteobacteria*. At the genus level, we observed a decrease in abundance of *Escherichia-Shigella*, *Akkermansia*, and *Veillonella*, and an increased abundance of *Prevotella*. To minimize the direct effects of glucocorticoid on the gut microbiota, patients received a low dose of oral prednisone (0.5–1 mg/kg/day) for 6 to 8 weeks, followed by a 4 week washout period before fecal sample collection. Therefore, the changes observed in the post-treatment group likely reflect a combination of the disease itself and the residual effects of glucocorticoid therapy.

## 5 Conclusion

Our study provides valuable insights into the gut microbiome in SAT. However, this study has several limitations. The cross-sectional design precludes determination of causal relationships between observed gut microbiota alterations and SAT pathogenesis or treatment response. Additionally, the relatively small sample size may limit the generalizability of our findings to larger populations. While we identified correlations between gut microbiota composition and SAT, the underlying mechanisms remain elusive. Future studies with larger cohorts and longitudinal designs are needed to establish causality and to track the dynamic changes in the gut microbiome throughout the course of SAT and treatment, and to explore the potential of modulating gut microbiome as a therapeutic intervention for this condition.
